# Structural Characterization of an α-D-glucan from *Bellamya purificata* and Its Protective Effects on Non-Alcoholic Fatty Liver Disease in Zebrafish

**DOI:** 10.3390/md24050159

**Published:** 2026-04-30

**Authors:** Xianhui Pan, Kangqi Zhou, Yuan Meng, Zhong Chen, Xuesong Du, Junqi Qin, Yong Lin, Tingjun Hu

**Affiliations:** 1 College of Animal Science and Technology, Guangxi University, Nanning 530003, China; panxhfisher@126.com; 2 Sean Key Laboratory of Comprehensive Exploitation and Utilization of Aquatic Germplasm Resources, Ministry of Agriculture and Rural Affairs, Key Laboratory of Aquaculture Genetic and Breeding and Healthy Aquaculture of Guangxi, Guangxi Academy of Fishery Sciences, Nanning 530021, China; zhoukqfisher@163.com (K.Z.); 15002381261@163.com (Y.M.); cz1050@163.com (Z.C.); gslnlxr@163.com (X.D.)

**Keywords:** *Bellamya purificata*, polysaccharide structure, NAFLD, gut microbiota

## Abstract

Non-alcoholic fatty liver disease (NAFLD) is a primary metabolic disorder that threatens adolescent health globally, with no effective therapeutic agents currently available. *Bellamya purificata* is a traditional Chinese medicine categorized as "medicinal food", and polysaccharides are among its active components. However, its physicochemical structure remains poorly characterized, and no study has evaluated its effects on NAFLD. In this study, a homogeneous neutral polysaccharide, α-D-glucan (Mw = 6412.704 kDa), was isolated from *B. purificata*. The structure of the polysaccharide was characterized using monosaccharide composition analysis, methylation analysis, NMR spectroscopy, and scanning electron microscopy. The backbone structure of the polysaccharide comprises →4)-α-D-Glcp-(1→ and →4,6)-α-D-Glcp-(1→, with side chains of α-D-Glcp-(1→ attached to the O-6 position of the 1→4,6)-α-D-Glcp-(1→ sugar residues. Additionally, QSPS-1D effectively reduced weight gain, hepatic lipid accumulation (TC and TG), and inflammatory responses (*tnf-α* and *il-1β*) in NAFLD zebrafish. Moreover, QSPS-1D alleviated dysbiosis by inhibiting harmful bacteria (e.g., *Stenotrophomonas*, *Agrobacterium*, and *Chryseobacterium*) and promoting beneficial microbiota (e.g., *Rothia*), which restored the Firmicutes-to-Bacteroidetes ratio. In parallel, it enhanced the expression of tight junction proteins (*zo-1* and *claudin-1*), leading to the repair of the intestinal mucosal barrier. These findings suggest that *B. purificata* polysaccharides may be a potential functional food for early NAFLD intervention, with effects potentially associated with the modulation of the gut microbiota.

## 1. Introduction

Non-alcoholic fatty liver disease (NAFLD) is one of the most prevalent chronic liver diseases globally, affecting 25–30% of the population [1]. Previous studies have shown that NAFLD is characterized by abnormal fat deposition in hepatocytes, which can progress to non-alcoholic steatohepatitis (NASH), liver fibrosis, cirrhosis, and even hepatocellular carcinoma (HCC), and is closely related to type 2 diabetes mellitus, cardiovascular disease, and other metabolic abnormalities in multiple systems [2]. Globally, 55.5% of patients with type 2 diabetes and 75.3% of obese individuals have NAFLD [3]. Notably, the prevalence of NAFLD among overweight or obese adolescents in China is 43% [4]. However, the challenging early diagnosis, significant disease heterogeneity, and absence of precise therapeutic targets in NAFLD mean that clinical treatment relies primarily on lifestyle interventions [5]. Therefore, the development of efficient, safe, and economical anti-NAFLD drugs is still imminent.

Shellfish polysaccharides, natural macromolecules with multiple biological activities, have garnered increasing attention in the medical community in recent years. Polysaccharides may therapeutically benefit NAFLD by regulating lipid metabolism, enhancing insulin sensitivity, reducing oxidative stress, and modulating the gut microbiota, among other mechanisms [6]. For instance, *Tegillarca granosa* polysaccharides effectively reduce excessive hepatic lipid accumulation, dyslipidemia, abnormal liver function, and steatosis in NAFLD mice, as well as enhance gut microbiota diversity and short-chain fatty acid content, thereby ameliorating dysbiosis [7]. Similarly, *Ostrea rivularis* polysaccharides modulated the gut microbiota composition in NAFLD mice, reducing the Firmicutes-to-Proteobacteria ratio, enhancing gut barrier function, and delaying NAFLD progression [8]. *The Bellamya purificata* snail, a species of gastropod in the family Viviparidae, is widely distributed in the natural waters of China, India, and parts of Africa [9]. As a typical freshwater shellfish, *B. purificata* is evolutionarily and biosynthetically related to marine shellfish; its glucans share similar structural features and bioactive potential with marine polysaccharides, making it a valuable extension of marine carbohydrate drug research. Snails have long been renowned in traditional Chinese medicine, and modern research has further confirmed that their extracts possess multiple bioactivities, including hepatoprotective and antibacterial properties [10,11,12]. Notably, recent studies have indicated that polysaccharides derived from gastropods have potential antiviral activity, improve alcoholic liver damage, and have antitumor effects [13,14,15]. Although polysaccharides from *B. purificata* are also regarded as bioactive, their specific structure, mechanism of action, and therapeutic potential for NAFLD remain unclear. This uncertainty severely limits their development and application.

Over the past few decades, zebrafish (*Danio rerio*) have been widely recognized as a viable model for studying numerous human diseases [16]. These fish exhibit high genetic and metabolic conservation with mammals, coupled with unique advantages such as low cultivation costs, rapid in vitro development, and optical transparency [17]. Meanwhile, the NAFLD model can be established in zebrafish by overfeeding them a high-calorie diet for five consecutive days, with disease progression resembling that observed in human patients [18]. Therefore, the zebrafish model offers novel insights into new drug evaluation and discovery, thereby advancing NAFLD research.

To address this, we isolated and purified a neutral polysaccharide component, QSPS-1D, from the muscle tissue of *B. purificata*. It was structurally characterized based on its monosaccharide composition and nuclear magnetic resonance (NMR). A NAFLD Zebrafish model was constructed to evaluate the ameliorative effect of QSPS-1D, and the main indexes were liver morphology, Oil Red O staining, and biochemical indexes. This study also analyzed the regulatory role of QSPS-1D in NAFLD using gut microbiota analysis. These findings provide scientific evidence to support early NAFLD prevention and the development of targeted therapeutic agents.

## 2. Results

### 2.1. Purification and Structural Characterization of QSPS-1D

**Figure 1:**
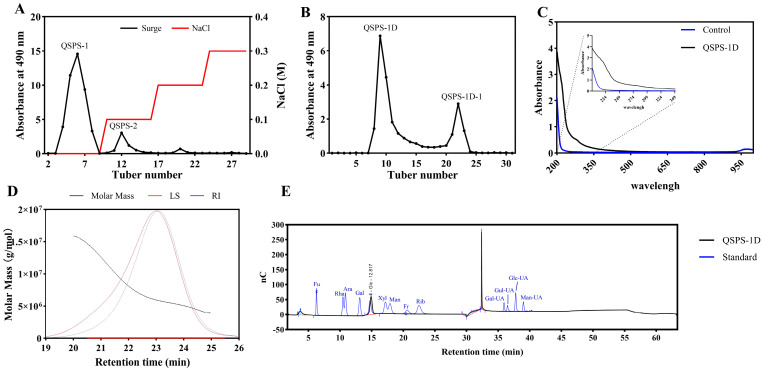
Preliminary structural characterization of QSPS-1D: Elution curves of polysaccharides from *Bellamya purificata* on (**A**) DEAE seplife FF column and (**B**) Sephacryl S-400HR column. (**C**) Ultraviolet spectrum of the neutral polysaccharide QSPS-1D. (**D**) HPLC distribution of neutral polysaccharides’ QSPS-1D. (**E**) Monosaccharide composition analysis of neutral polysaccharides’ QSPS-1D.

In this study, the crude polysaccharide extraction rate was 15.7%, and the total carbohydrate content was 25.6% in dried snail meat using aqueous-alcoholic precipitation. Ion-exchange chromatography was performed based on the differential charge of the polysaccharides. Acidic polysaccharides formed strong electrostatic interactions with the resin and exhibited longer retention times, whereas neutral polysaccharides with weaker interactions were eluted first. This allowed the separation of two fractions, QSPS-1 and QSPS-2, which accounted for 64.2% and 10.8% of the total polysaccharide content, respectively. Given its higher polysaccharide content, QSPS-1 was selected for further purification by gel filtration chromatography, which separates molecules based on their size and spatial conformation. In this process, larger molecules are excluded from the gel pores and eluted first, whereas smaller molecules permeate the gel and are retained for a longer time. The gel chromatogram revealed two peaks (QSPS-1D and QSPS-1D-1), which corresponded to different molecular weight fractions of the same polysaccharide, likely due to molecular chain aggregation or conformational differences rather than incomplete separation or residual contamination from QSPS-2. The total polysaccharide contents of QSPS-1D and QSPS-1D-1 were determined to be 97.0% and 38.3%, respectively (Figure 1A,B). Based on these results, the QSPS-1D was selected for further studies. The UV results indicated that no distinct absorption bands were observed for QSPS-1D at 260 nm (indicative of nucleic acids) or 280 nm (indicative of proteins). Furthermore, the absence of any absorption bands in the 210–215 nm region confirmed that the substance did not contain uronic acid residues (Figure 1C). A single narrow peak observed in the high-performance gel filtration chromatography (HPGFC) chromatogram indicated the relative homogeneity and purity of QSPS-1D. The weight-average molecular weight (Mw), number-average molecular weight (Mn), and polydispersity index (Mw/Mn) were 6,412.704 kDa, 6,039.582 kDa, and 1.062, respectively (Figure 1D and Table S1). Moreover, high-performance anion-exchange chromatography (HPAEC) chromatograms and mixing standards showed that QSPS-1D contained only glucose (Figure 1E).

### 2.2. FT-IT and GC-MS Analysis of QSPS-1D

**Figure 2:**
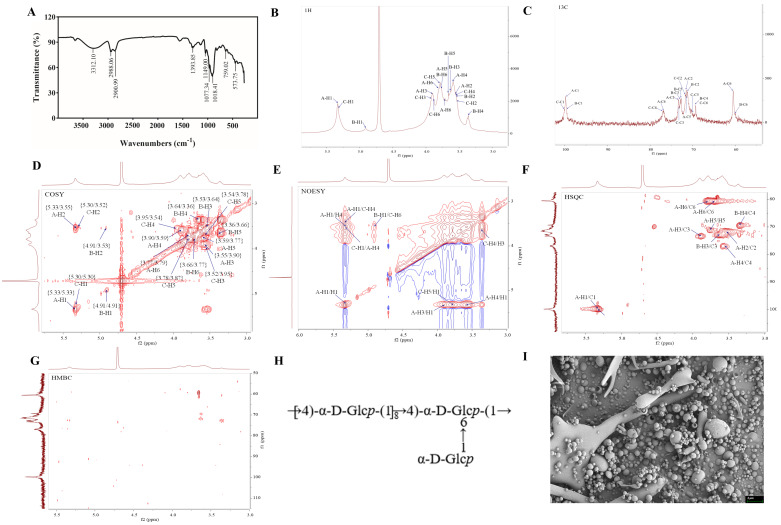
FI-IR and NMR spectra of QSPS-1D. (**A**) FI-IR spectrum of QSPS-1D after methylation; (**B**) 1H NMR; (**C**) 13C NMR; (**D**) 1H-1H COSY; (**E**) 1H-1H NOESY; (**F**) 1H-13C HSQC; (**G**) 1H-13C HMBC; (**H**) predicted chemical structure of QSPS-1D; (**I**) SEM images (2 μm) of QSPS-1D.

The polysaccharide resorption bands of QSPS-1D were analyzed using FT-IT spectroscopy (Figure 2A). The results showed that the absorption bands between 3600 and 3200 cm^−1^ were the telescopic vibration absorption bands of -OH and at 3312.10 cm^−1^ were the telescopic vibration absorption bands of O-H, which are characteristic bands of sugar. The absorption bands at 2988.06 cm^−1^ and 1018.41 cm^−1^ are attributed to the C-H stretching vibration and C-O stretching vibration, respectively. The weak absorption band at 929.01 cm^−1^ is attributed to the asymmetric ring-stretching vibration of D-glucopyranose [19]. The overall spectral features indicate that QSPS-1D has a typical polysaccharide structure, as previously reported.

Comparison was made with a spectral database of partially methylated alditol acetates (PMAAs) from the Complex Carbohydrate Research Centre (CCRC). The identification of PMAAs based on retention time and typical fragment ions is presented in Table 1. The GC-MS chromatogram in this study showed that QSPS-1D was primarily composed of four types of glucopyranosyl residues, including t-Glcp(1→, →4)-Glcp(1→, →3,4)-Glcp(1→, and →4,6)-Glcp(1→, with molar ratios of 12.32:77.17:1.08:9.43. This indicates that the branching point (3,4,6-linked α-Glc) in QSPS-1D accounts for 22.83 % of the total glycosyl residues, suggesting that the polysaccharide has a highly branched backbone.

### 2.3. NMR Analysis of QSPS-1D

The structure of QSPS-1D was further characterized by NMR spectroscopy. In the 1H NMR spectrum, the signals of QSPS-1D were concentrated between δ 3.0–5.5 ppm, and multiple coupled signal peaks (e.g., δ 4.91, 5.3,and 5.33 ppm) were recognized in the δ 4.3–5.4 ppm heteroheader signal region, indicating that the sample was in α-glycosidic bond conformation and contained multiple sugar residues, which was consistent with the FT-IR results [20] (Figure 2B). The non-isohead hydrogen signals were mainly concentrated in the δ 3.1–4.2 ppm region, and the individual signals needed to be combined with COSY and HSQC spectra to attribute the H2-H6 chemical shifts of each sugar residue separately due to the severe overlap (Figure 2C). At δ = 4.71 ppm, a strong signal corresponding to the solvent peak was observed. Combining the ^13^C NMR and HSQC spectra, the cross-peak signals at δ 5.33/99.8, 4.91/98.26, and 5.3/100.1 ppm correspond to H1/C1 belonging to the four sugar residues (A, B, and C), which are all α-configuration glucose residues (Figure 2D).

The chemical shift results for 1H and 13C are presented in Table 2. In brief, the chemical shifts of hydrogen on the sugar rings of three sugar residues were completed by COSY spectral results, including H2-H6 of sugar residues A/B/C (3.55, 3.9, 3.59, 3.77, and 3.79, 3.71 ppm; 3.53, 3.64, 3.36, 3.66, and 3.77 ppm; 3.52, 3.95, 3.54, 3.78, and 3.87 ppm) (Figure 2E). The chemical shift attribution of C on the sugar ring was completed based on HSQC signals (Figure 2D). The chemical shifts in C1 and C4 of sugar residue A, C1 of sugar residue B, and C1, C4, and C6 of sugar residue C were shifted towards the lower field, indicating that these residues underwent substitutions to varying extents at positions O-1, O-4, and O-6 of the sugar ring. However, the weaker signal in sugar residue D only completed the chemical shift in C on the sugar rings of C1 and C2; therefore, the low-field shift in C was not observed. Finally, this study combined the results of methylation analysis, heterozygous signals, and literature reports to deduce that the sugar residues A to C were →4)-α-D-Glcp(1→, α-D-Glcp(1→, and →4,6)-α-D-Glcp(1→, respectively [21,22,23].

The NOESY spectrum showed key cross-peak signals at δ5.33/3.59 (A-H1/A-H4), δ5.33/3.54 (A-H1/C-H4), δ4.91/3.87 (B-H1/C-H6), and δ5.3/3.59 (A-H4/C-H1), which further confirmed the glycosyl bonding of the above residues (Figure 2F). In summary, the combination of NMR data and methylation analysis revealed that the backbone of QSPS-1D is formed by the linkage of A (→4)-α-D-Glcp-(1→) and C (→4,6)-α-D-Glcp-(1→) sugar residues, while the side chains consist of B (α-D-Glcp-(1→) attached to the O-6 position of the C (→4,6)-α-D-Glcp-(1→) sugar residues. The putative structure of the QSPS-1D repeat unit is shown in Figure 2H.

The purified QSPS-1D morphology was examined using scanning electron microscopy, which revealed spherical structures with smooth surfaces, irregular granularity, and varying particle sizes (Figure 2I). This information is valuable for understanding the physicochemical properties of polysaccharides and their applications as biomaterial.

### 2.4. Effects of QSPS-1D on Growth and Liver Morphology

**Figure 3:**
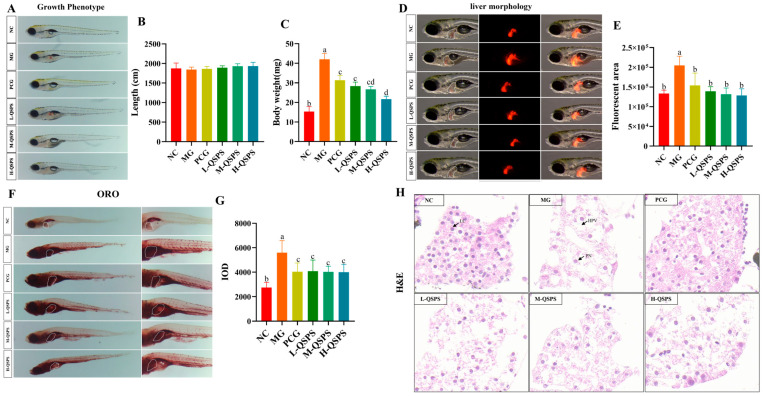
QSPS-1D alleviated high-fat-diet-induced weight gain and hepatic damage. (**A**) Growth phenotype; (**B**) body length; (**C**) body weight; (**D**) liver morphology; (**E**) liver fluorescence area; (**F**) hepatic fat deposition; (**G**) liver red oil index; (**H**) liver histological analysis (20 μm); HPV, hepatocyte vacuoles; PN, hepatocellular nuclear shift; LC, hepatic cell; different letters indicate significant differences (*p* < 0.05), while letters sharing the same symbol denote no statistical difference between the groups. The same applies below.

As shown in Figure 3A,B, there was no remarkable difference in the body lengths of zebrafish between the different treatment groups. Significant differences in body weight and liver morphology between the control and model groups confirmed the success of the high-fat modeling. Additionally, increasing the polysaccharide concentration led to a notable decrease in zebrafish body weight and a reduction in liver fluorescence area (Figure 3C–E). Moreover, a representative pathomicrograph of the zebrafish liver showed that the model group had abnormal hepatocyte structures, a large number of vacuoles in the liver, deformed and irregularly arranged hepatocytes, and severe lipid droplet deposition compared with the control group. However, compared to the model group, QSPS-1D-fed and etimeber-treated zebrafish showed notably reduced vacuolization of hepatocytes, inhibited lipid accumulation, and eliminated inflammation, implying a reversal of liver injury (Figure 3F–H). In addition, there was a clear difference in the mitigating effect of 80 µg/mL QSPS-1D on HFD-induced liver injury.

### 2.5. QSPS-1D Improved Aberrant Biochemical Parameters and Hepatic Inflammation

**Figure 4:**
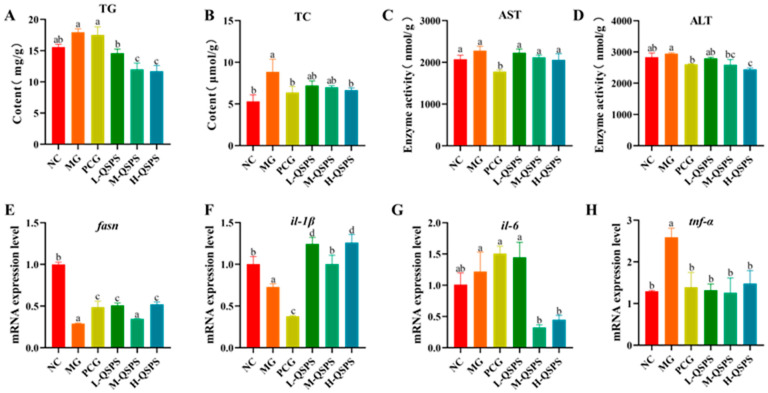
QSPS-1D alleviates high-fat-diet-induced adipogenesis and inflammation. (**A**,**B**) Hepatic TC and TG levels; (**C**,**D**) hepatic AST and ALT levels; (**E**) *fasn* gene expression levels in the liver; and (**F**–**H**) inflammatory gene expression levels of *il-1β*, *il-6*, and *tnf-α* in the liver. AST, aspartate aminotransferase; ALT, alanine aminotransferase; TC, total cholesterol; TG, triglyceride. Different letters indicate significant differences (*p* < 0.05), while letters sharing the same symbol denote no statistical difference between the groups. The same applies below.

The model group showed significantly higher total cholesterol (TC) and triglyceride (TG) concentrations in the zebrafish liver than the control group, suggesting abnormalities in lipid metabolism. All QSPS-1D doses showed good inhibition of lipid accumulation. Among them, 40 and 80 μg/mL QSPS-1D had similar abilities to reduce TC and TG levels in fish liver. Several enzymes in the liver are recognized as markers of early liver injury (Figure 4A,B). Zebrafish liver alanine aminotransferase (ALT) and aspartate aminotransferase (AST) activities were significantly elevated in the model group compared to the control group, suggesting hepatocellular injury (Figure 4C,D). Notably, the inhibitory effect of 80 μg/mL on the elevation of hepatic ALT and AST levels was stronger than that of the other concentrations, especially for ALT removal. In addition, the hepatic *tnf-α* and *il-1β* levels were significantly elevated and *il-6* and *fasn* levels were reduced in the model group compared with the control group, but their expression levels were markedly improved after QSPS-1D and Etimeber supplementation (Figure 4E–H). Interestingly, all three doses of QSPS-1D ameliorated hepatic fat accumulation and inhibited inflammation, indicating the potential hepatoprotective and anti-inflammatory effects of QSPS-1D in HFD-induced zebrafish models.

### 2.6. QSPS-1D Restores Healthy Gut Morphology and Barrier Function

**Figure 5:**
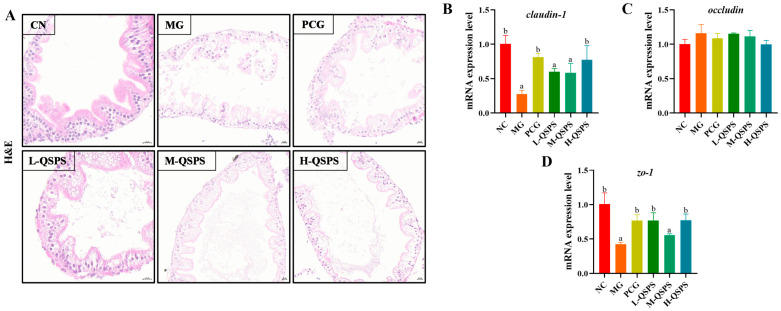
QSPS-1D treatment improved intestinal barrier function. (**A**) Intestinal histological analysis (20 μm). (**B**–**D**) Intestinal claudin-1, occludin and zo-1 gene expression levels. Different letters indicate significant differences (*p* < 0.05). The same letters indicate no significant differences (*p* > 0.05).

Zebrafish were subjected to histopathological examination of the gut using hematoxylin and eosin (H&E) staining (Figure 5A). Mild intestinal damage was observed in the model group, which was mainly manifested by necrosis and detachment of some epithelial cells, loosely arranged connective tissue, and mild submucosal capillary dilatation. Notably, we observed a dose–response relationship between QSPS-1D intervention and alleviation of HFD-induced intestinal damage. The guts of QSPS-1D-treated zebrafish had distinct and intact mucosal structures, without any obvious inflammatory changes. Therefore, we hypothesized that QSPS-1D treatment might repair HFD-induced intestinal morphological damage by enhancing intestinal nutrient absorption and promoting intestinal development. RT-qPCR analysis showed that the mRNA expression levels of *zo-1* and *claudin-1* in the zebrafish gut were significantly decreased after high-fat diet induction, suggesting a significant decrease in intestinal barrier integrity, which was effectively reversed by all three doses of QSPS-1D intervention (Figure 5B–D). Overall, QSPS-1D may play a critical role in maintaining intestinal homeostasis in zebrafish fed a high-fat diet.

### 2.7. QSPS-1D Restored the Abundance of Beneficial Gut Flora

**Figure 6:**
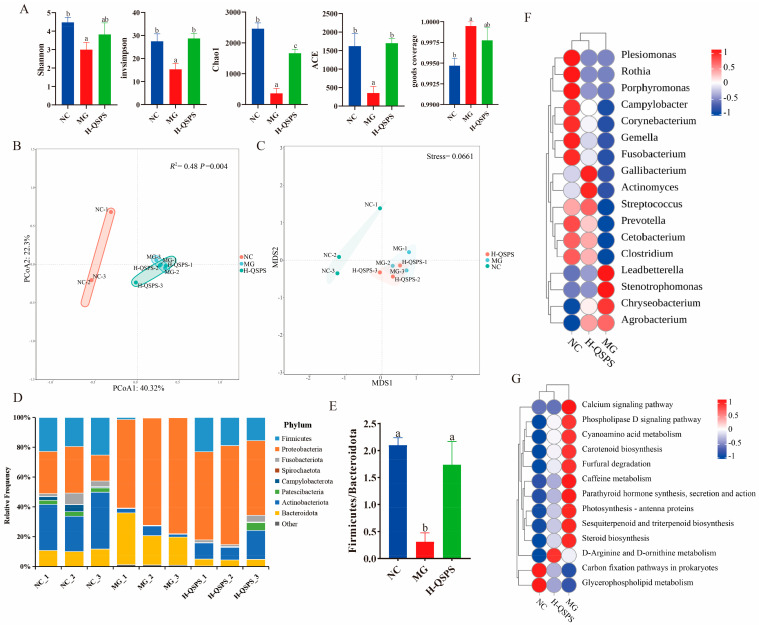
QSPS-1D treatment enhances the diversity and structure of gut microbiota. (**A**) α-diversity indices for Shannon, invSimpson, Chao1, ACE, and goods coverage; (**B**) PCoA based on Bray–Curtis distance differences; (**C**) unweighted UniFrac-based NMDS; (**D**) relative abundance of gut microbiota at the phylum level; (**E**) ratio of Firmicutes to Bacteroidetes; (**F**) heatmap analysis at the genus level; (**G**) microbial function prediction based on PICRUSt2. Different letters indicate significant differences (*p* < 0.05). The same letters indicate no significant differences (*p* > 0.05).

Differences in the results of α-diversity analyses between the groups are shown in Figure 6, which mainly showed that the Shannon, invsimpson, Chao1, Ace, and good coverage indices of gut microbes were dramatically reduced (*p* < 0.05) after high-fat feeding in the MG group; however, these changes could be markedly ameliorated by the intake of H-QSPS (Figure 6A). PCoA based on Bray–Curtis distance differences identified a high degree of variability between the groups, particularly a significant clustering between the NC and MG groups, which is consistent with the results of the NMDS analyses. Adonis analysis showed a major difference between the groups (*R*^2^ = 0.48, *p* = 0.004), confirming the large contribution of high-fat or H-QSPS intervention to the significant difference (Figure 6B,C).

At the phylum level, the gut microbes in all groups consisted mainly of Actinobacteriota, Bacteroidota, and Firmicutes, with some variation between the groups (Figure 4D). Specifically, the relative abundances of Actinobacteriota and Bacteroidota were significantly higher in the MG group after high-fat intake, whereas that of Firmicutes was significantly lower in the MG group. The Firmicutes/Bacteroidota (F/B) ratio was dramatically reduced in the MG group compared to the control group and markedly increased after H-QSPS intervention (*p* < 0.05) (Figure 6E). Heatmap analysis revealed significant differences in bacterial taxa at the genus level between the NC groups. In particular, the MG group showed a substantial increase in the relative abundance of four genera: *Stenotrophomonas*, *Chryseobacterium*, *Leadbetterella*, and *Agrobacterium* (Figure 6F). Notably, this microbial shift was effectively counteracted by the H-QSPS intervention. We further analyzed the functional characteristics of gut microbes in each group using PICRUSt2. Compared with the MG group, the H-QSPS group clustered with the NC group as a unit in the level3 KEGG pathways. Compared with the NC group, the metabolic functions of the MG group may have been disturbed, including the significantly increased expression levels of steroid biosynthesis, cyanoamino acid metabolism, D-arginine, and D-ornithine metabolism, which were effectively restored after H-QSPS treatment (Figure 6G). Overall, H-QSPS administration can potentially alleviate the problem of high-fat-diet-induced intestinal flora imbalances.

### 2.8. Correlation Analysis of Biochemical Indicators with Microbiome

**Figure 7:**
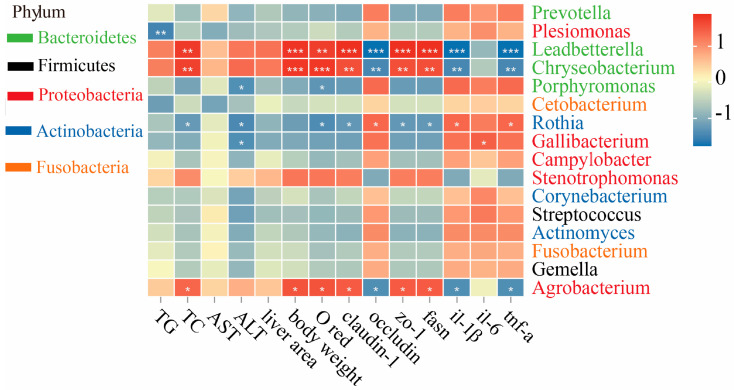
Correlation analysis between biochemical indicators and the microbiome was performed using Spearman’s correlation analysis. Red indicates a positive correlation, and blue indicates a negative correlation. * represents *p* < 0.05; ** represents *p* < 0.01; *** represents *p* < 0.001.

Fourteen indicators were selected for the Mantel test analysis with the gut microbiota at the genus level to study their relationship (Figure 7). This result showed that Proteobacteria (including *Leadbetterella*, *Chryseobacterium*, and *Agrobacterium*) were positively correlated with TC, IOD, *claudin-1*, *zo-1*, *fasn*, and body weight indexes, but negatively correlated with *il-1β*, *tnf-α*, and *occludin* (*p* < 0.05 or *p* < 0.01). However, opposite results were observed for *Rothia* in Actinobacteria (*p* < 0.05).

## 3. Discussion

Freshwater snails are valuable medicinal and edible species that have been featured in the Chinese Pharmacopoeia for over two millennia, with polysaccharides being one of their key bioactive constituents. To date, several glucans have been reported from closely related species, yet their structural features remain incompletely characterized or inconsistent. For instance, two polysaccharides extracted and isolated from *C. chinensis* exhibited immunomodulatory activity, with a molecular weight of 226–235 kDa and a backbone composed of (1→3)-α-D-Glc linkages [15]. However, as noted in a critical assessment, the presence of α-1,3 linkages in those polysaccharides remains ambiguous due to insufficient methylation and NMR evidence, and the reported sulfated glucan from the same species lacked detailed linkage analysis and sulfate substitution patterns [12]. Another study reported two glucans (BPS-1 and BPS-2) extracted from *B. purificata*, both exhibiting anti-inflammatory activity, with molecular weights of 7,200 and 8,300 kDa, respectively, and a structural backbone primarily composed of (1→4)-α-D-Glc, (1→3)-α-D-Glc, and (1→6)-α-D-Glc [11]. Nevertheless, the extremely high molecular weights (exceeding 7,000 kDa) suggest possible aggregation or incomplete purification, and the presence of (1→3)-linkages was not unequivocally confirmed by methylation or NMR data. In contrast, the present study provides a well-characterized neutral glucan (QSPS-1D) from *B. purificata* muscle tissue, obtained via a mild hot-water extraction and rigorous purification protocol. Our methylation analysis and NMR spectroscopy consistently indicate that QSPS-1D is a high-molecular-weight (6,412.704 kDa) α-D-glucan, whose backbone consists of →4)-α-D-Glc-(1→ and →4,6)-α-D-Glc-(1→. However, due to the low content of branches and terminal sugars, and based on the available NMR results, there appears to be only one α-D-Glc-(1→ side chain within the repeating unit. This requires further analysis using a debranching enzyme (such as pullulanase) to determine the exact length of the side chain. Importantly, no convincing evidence for (1→3)-linkages was found in our study, which distinguishes QSPS-1D from the previously reported but structurally ambiguous glucans [11,15]. Moreover, unlike the sulfated and uronic acid-containing polysaccharides described in *B. quadrata* and *C. chinensis* [12,13,24]. Thus, the discovery of QSPS-1D further highlights the structural diversity within the genus Bellamya.

Subsequently, no distinct bands were detected in the Fourier-transform infrared (FT-IR) spectrum in the range of 1600–1650 cm^−1^. This phenomenon can be partly ascribed to the absence of uronic acids in QSPS-1D, which is consistent with the monosaccharide composition results of this study. Moreover, the drying degree of the polysaccharide samples, the pressing conditions during sample preparation, and ambient humidity can all affect the intensity of the water band in this region. In this study, the FT-IR samples were thoroughly vacuum-dried before testing, effectively reducing the content of bound water in the samples; this may also be another factor contributing to the lack of distinct absorption peaks in this region. Similar phenomena have been frequently reported in the literature for neutral polysaccharides such as *Poria cocos* and *Chinese yam* polysaccharides [25,26]. Collectively, these findings suggest that QSPS-1D exhibits characteristics that are highly consistent with those of neutral polysaccharides.

Furthermore, the scanning electron microscopy (SEM) images of QSPS-1D in this study revealed regular circular particles of varying sizes, which is similar to the morphology of vacuum-dried polysaccharides reported in the literature. Previous research has demonstrated that the SEM morphology of polysaccharides is not fixed but is influenced by various factors, with the drying method being one of the most crucial factors. Specifically, for samples of the same *Auricularia auricular* polysaccharide, those dried by vacuum drying exhibited “smooth-surfaced spherical particles,” while freeze-dried samples presented “continuous, porous, flaky structures with a loose texture” [27]. This is primarily due to the slow evaporation of the solvent under vacuum conditions, allowing polysaccharide molecules sufficient time to undergo ordered intra- and intermolecular arrangements, forming low-energy spherical structures to minimize surface energy, thereby resulting in relatively regular spherical or granular shapes. The SEM morphology of polysaccharides reflects their microstructure in the solid state rather than a direct representation of their solution conformation. Therefore, a comprehensive evaluation of the *B. purificata* polysaccharide structure can be performed by combining solution conformation analysis techniques, such as dynamic light scattering (DLS) and size-exclusion chromatography coupled with multi-angle laser light scattering (SEC-MALLS).

Current research generally agrees that α-D-glucan is an α-glucan primarily formed through α1→6 and/or α1→4 linkages. It can be widely applied in the food, pharmaceutical, and other fields, presenting broad prospects for development and utilization. For instance, *C. chinensis* polysaccharide (CCPS), whose main chain consists of (1→3)-α-D-Glc units, can alleviate the progression of atherosclerotic plaques (AS) by lowering blood lipid levels and modulating the gut microbiota [24]. In parallel, Huangjiu polysaccharides, featuring a backbone of α-D-Glc units linked via (1→4) bonds, effectively enhance intestinal barrier function, reduce serum and hepatic lipopolysaccharide levels, and ameliorate ethanol-induced hepatic dysfunction and steatosis [28]. *Radix Puerariae thomsoni* polysaccharides, with a main chain of α-D-1,3-glucan, markedly mitigates high-fat-diet-induced liver injury by suppressing inflammatory responses, improving glucose metabolism, and reducing steatosis, thereby alleviating NAFLD symptoms [29]. Freshwater snail polysaccharides have demonstrated promising effects in combating hepatitis B, alleviating ankylosing spondylitis, and mitigating alcoholic liver disease. However, research on their application in NAFLD remains unreported and warrants further expansion. In this context, the present study isolated a novel polysaccharide, QSPS-1D, which exhibits structural similarities to previously reported compounds speculated to possess potential therapeutic utility for NAFLD.

Thereafter, we employed a high-fat-diet-induced wild-type AB zebrafish model of NAFLD to investigate the underlying mechanisms of action of QSPS-1D. This study demonstrated that QSPS-1D had no effect on body length in the NAFLD zebrafish model but effectively reduced body weight and hepatic fluorescent area, indicating that QSPS-1D can regulate weight gain and lipid accumulation without compromising the developmental growth of fish. Notably, QSPS-1D treatment resulted in a dose-dependent reduction in histopathological damage, including diminished hepatocyte vacuolation, improved cellular alignment, and decreased liver lipid droplet deposition. These morphological improvements were accompanied by significant decreases in total cholesterol (TC) and triglyceride (TG) levels, indicating the restoration of lipid metabolic homeostasis [30]. Notably, the 80 μg/mL dose demonstrated exceptional efficacy in alleviating elevated ALT and AST levels (markers of hepatocyte damage). This indicates potent hepatoprotective effects at higher concentrations, potentially achieved through enhanced membrane stability and cellular defense mechanisms [31]. It is important to note that the doses of QSPS-1D used in this study (20–80 μg/mL) were comparatively high when compared with the egg yolk powder control (100 μg/mL). Although these doses effectively mitigated non-alcoholic fatty liver disease (NAFLD)-like pathology in zebrafish, the translational applicability to mammalian systems necessitates further verification. Future research should establish a dose–response curve and assess the therapeutic window of QSPS-1D in a more clinically relevant model, such as rodents. Notwithstanding, the current findings offer proof-of-concept evidence indicating that QSPS-1D exhibits potential hepatoprotective activity under the tested conditions.

Present studies have generally recognized that intestinal barrier dysfunction is closely associated with NAFLD severity [32]. In addition, transmembrane proteins (e.g., *claudins-1* and *occludin*) and cytoplasmic scaffold proteins (such as *zo-1*) serve as key components of the intestinal mechanical barrier, playing vital roles in maintaining the polarity of intestinal epithelial cells and regulating the selective permeability of the barrier [33]. This study revealed that high-fat feeding induces intestinal structural abnormalities in zebrafish, concurrently suppressing *zo-1* and *claudin-1*. In contrast, QSPS-1D treatment markedly alleviated these pathological changes in the liver. Moreover, histological sections revealed that the intestinal mucosal structure in QSPS-1D-treated zebrafish was more intact than that in the model group. Additionally, compromised intestinal barrier function typically activates downstream proinflammatory cascades [34]. In this study, a high-fat diet dramatically increased hepatic *tnf-α* and *il-1β* levels, which were effectively suppressed by QSPS-1D. Similarly, α-D-glucan isolated from Huangjiu restored the mucosal barrier and re-established intestinal immune homeostasis in ALD mice, as manifested by the significantly upregulated expression of tight junction proteins (e.g., *zo-1*, *occludin*, *and claudin-1*) alongside reduced hepatic pro-inflammatory cytokine levels [28]. In summary, QSPS-1D may exert beneficial effects by repairing the intestinal barrier and suppressing inflammation in the liver.

The gut microbiota plays a pivotal role in maintaining host immune homeostasis and metabolic health, as well as disrupting intestinal barrier function, triggering low-grade inflammation, and driving the development of NAFLD [35,36]. The Firmicutes-to-Bacteroidetes ratio (F/B ratio) is widely used as a macroscopic indicator of gut microbial homeostasis [37]. This study confirms that QSPS-1D is remarkably effective in reversing the loss of diversity and structural disruption of the zebrafish gut microbiota induced by a high-fat diet. Interestingly, unlike the reduced F/B ratio typically observed in classical mammalian NAFLD models, this study revealed a distinctive microbial response pattern in diseased zebrafish larvae: an elevated F/B ratio manifested as an increased relative abundance of Firmicutes and a decreased abundance of Bacteroidetes. This divergence likely stems from species-specific differences. In mammals, NAFLD is typically associated with a decreased F/B ratio, which facilitates enhanced energy harvest from the diet and promotes hepatic lipid accumulation [38]. Nevertheless, the gut microbiota composition of zebrafish exhibits significant disparities compared with that of mammals. Significantly, it has been proven that when germ-free zebrafish are colonized with mouse gut microbiota, the relative abundance of bacterial lineages undergoes a shift to approximate the normal composition of the recipient zebrafish host, rather than preserving the donor’s microbial profile [39]. This finding emphasizes that host-specific factors play a fundamental role in shaping the gut microbial community structure. The developmental stage of the model organism is another crucial factor influencing gut microbiota composition. Prior research has shown that the gut microbiota of healthy juvenile zebrafish is primarily composed of the phylum Firmicutes, with a relatively low proportion of Bacteroidetes [40,41]. In the larval zebrafish model employed in this study, the decreased F/B ratio in the high-fat diet group is regarded as an indication of severe dysbiosis. In contrast, QSPS-1D intervention effectively restored the gut microbiota to the expected healthy baseline, highlighting its strong microbiota-remodeling ability. Consistently, Jiuzao polysaccharides were also discovered to mitigate ethanol-induced liver damage in juvenile zebrafish by augmenting the abundance of Proteobacteria and Firmicutes and reducing the abundance of Bacteroidetes [42]. Additionally, a decrease in Firmicutes abundance has been associated with high sugar diets and lipopolysaccharide production in humans and other mammals [43,44].

An in-depth analysis at the genus level provides a robust mechanistic explanation for these macro-level changes. We observed that opportunistic pathogens *Stenotrophomonas* and *Agrobacterium*, belonging to the phylum Proteobacteria (recognized as inflammation-associated phyla), along with *Chryseobacterium* from the phylum Bacteroidetes, were significantly enriched in the NAFLD model group. The proliferation of these genera is closely associated with systemic infection and intestinal inflammation, and their abundance exacerbates intestinal barrier damage and increases endotoxin production [45]. The microbiome-phenotype association analysis in this study provides a crucial link: these harmful bacterial genera showed significant positive correlations with TC, TG, *fasn*, *claudin-1*, *zo-1*, and body mass index, and negative correlations with *occludin*. This suggests that overgrowth is accompanied by impaired intestinal barrier function, potentially facilitating the translocation of harmful substances [45]. Conversely, their suppression by QSPS-1D aids in restoring gut health and alleviating the antigenic load in the liver. Simultaneously, we observed a marked increase in *Rothia* abundance following polysaccharide treatment. This bacterium exhibits notably reduced abundance in the intestines of obese children, consistent with the findings in the model group of this study [46]. Correlation analysis further revealed strong negative correlations between this genus and TC, IOD, claudin-1, *zo-1*, *fasn*, and body mass index, implying that the increased presence of *Rothia* may effectively mitigate the adverse effects of a high-fat diet. PICRUSt analysis indicated that treatment with QSPS-1D led to the enrichment of multiple pathways, such as lipid metabolism and amino acid metabolism. Significantly, the upregulation of carbon fixation pathways in prokaryotes implies that QSPS-1D may enhance the metabolic capacity of beneficial gut bacteria, thereby promoting the production of short-chain fatty acids (SCFAs) from dietary carbohydrates [47]. Moreover, a notable upregulation of the glycerophospholipid metabolic pathway was detected in the QSPS-1D group. As a core node in phospholipid metabolism, this pathway is crucial for maintaining cell membrane integrity, facilitating lipid transport, and mediating signal transduction [48]. Consistent with this result, research on *Mori Fructus* polysaccharides has shown that the modulation of glycerophospholipid metabolism can effectively alleviate lipid metabolism disorders in mice with alcohol-induced liver injury [49]. Nevertheless, the high molecular weight of QSPS-1D (6412.704 kDa) in this study may impede its direct absorption in the gastrointestinal tract. However, substantial evidence suggests that high-molecular-weight polysaccharides are metabolized by gut bacteria into SCFAs and other bioactive metabolites, which then enter the bloodstream and regulate hepatic lipid metabolism [50]. Therefore, the hepatoprotective effects of QSPS-1D are likely mediated via gut microbiota-dependent mechanisms rather than direct uptake by the liver. Future pharmacokinetic and metabolomic studies are required to trace the metabolic pathways of QSPS-1D and identify the active metabolites responsible for its anti-NAFLD activity.

**Figure 8:**
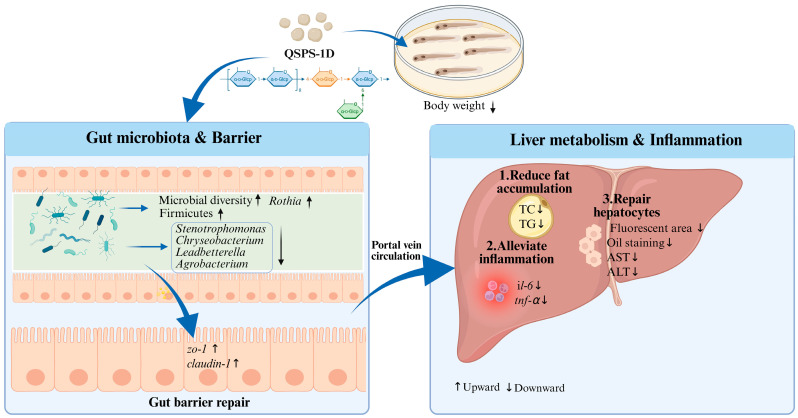
Mechanism of QSPS-1D in mitigating NAFLD damage by regulating gut microbiota, hepatic lipid metabolism, and inflammatory responses. AST, Aspartate aminotransferase; ALT, Alanine aminotransferase; TC, Total cholesterol; TG, Triglyceride. ↑ indicates an increase. ↓ indicates a decrease.

In summary, this study successfully isolated a structurally uniform natural neutral polysaccharide (α-D-glucan) from *B. purificata*. We also verified that it had no significant impact on the growth of zebrafish with high-fat-diet-induced NAFLD, while effectively improving liver and intestinal health in the fish. On the one hand, QSPS-1D may be broken down into short-chain fatty acids or other bioactive metabolites, thereby improving intestinal morphology and increasing the relative abundance of beneficial gut microbiota. It then enters the bloodstream and reaches the liver, where it enhances the liver’s anti-inflammatory activity and lipid metabolism to repair the liver structure and reduce abnormal lipid deposition (Figure 8).

## 4. Materials and Methods

### 4.1. Materials and Reagents

*B. purificata* was obtained from the breeding base of Guangxi Academy of Fishery Sciences (Nanning, China). Sephacryl S-400HR and DEAE seplife FF were purchased from the GE Healthcare Ltd. (Stockholm, Sweden). Glucose, Papain, dimethyl sulfoxide, sodium borodeuteride, and acetic anhydride were purchased from MERCK (Shanghai, China). Sodium nitrate was purchased from Sinopharm (Beijing, China) and was of AR grade. Trifluoroacetic acid, acetic acid, and dichloromethane were purchased from CNW Technologies GmbH (Shanghai, China). Ezetimibe was purchased from AdooQ Bioscience. (Nanning, China), and zebrafish embryo E3 culture medium was purchased from Shanghai Fish Bio Co., Ltd. (Shanghai, China). Triglyceride (TG), total cholesterol (TC), alanine aminotransferase (ALT), and aspartate aminotransferase (AST) assay kits were obtained from the Nanjing Jianjian Bioengineering Institute (Nanjing, China). HiPure Stool DNA Kit (Magen, Guangzhou, China). High-fat egg yolk powder and 1xE3 zebrafish culture solution were provided by Suzhoeau Muri Biotech Co., Ltd. (Suzhou, China).

### 4.2. Isolation, Purification and Characterization of QSPS-1D

#### 4.2.1. Isolation and Purification

A crude polysaccharide extract was obtained from *B. purificata* using water extraction and ethanol precipitation methods, followed by determination of its purity using the sulfuric acid-phenol method. Briefly, after dissection, the gastropedal muscle of the snail was isolated, dried, and pulverized. Sifting through a 60-mesh sieve yielded a grayish-white powder. Next, 100 g of the sample was mixed with fivefold volumes of anhydrous ethanol to remove lipids and other low-molecular-weight substances. Distilled water was then added to the precipitate at a 1:30 ratio. After incubation in a 60 °C water bath for 4 h, the mixture was centrifuged at 6000× *g* for 10 min to collect the supernatant. The supernatant was concentrated, and fourfold volumes of anhydrous ethanol were then added. After 12 h of precipitation, the samples were centrifuged at 8000× *g* for 10 min, and the crude water extract was collected for further analysis. Subsequently, the dried crude water extracts were dissolved in water and deproteinized using the Sevag method. Petroleum ether was used to remove fat, and macroporous resin AB-8 was used to remove the pigment. The solution was dialyzed (3000 Da) against water, concentrated, and freeze-dried to obtain crude polysaccharides.

The sample was applied to a DEAE-cellulose column (26 × 400 mm) and sequentially eluted with distilled water at 4 mL/min, followed by 0.1, 0.2, and 0.3 M NaCl. The eluents of the same peak were combined, concentrated under reduced pressure, dialyzed, and lyophilized to obtain QSPS-1 and QSPS-2 polysaccharide fractions (Figure 1A). The major polysaccharide fraction of QSPS-1 in the aqueous eluate was further purified using a gel chromatography column (26 × 1000 mm) packed with Sephacryl S-400HR (Stockholm, Sweden). The purified polysaccharide of the core QSPS-1D was obtained by vacuum drying. Each fraction was collected and assayed for carbohydrate content using the phenol–sulfuric acid method at 490 nm wavelength. Briefly, a standard solution with a concentration gradient is prepared by sequentially diluting a 100 μg/mL glucose stock solution with distilled water to plot a standard curve. Then, 2–5 mg of the sample is weighed, 1 mL of sterile water is added, and the mixture is shaken to homogenize. After centrifugation at 12,000 rpm for 10 minutes, the supernatant is diluted with distilled water to a concentration of 50 μg/mL. Transfer 0.1 mL of the supernatant to a test tube, add 50 μL of 5% phenol solution followed by 500 μL of concentrated sulfuric acid, mix well, and allow the mixture to stand in the dark for 10 minutes. Measure the absorbance at 490 nm and use the standard curve to calculate the polysaccharide content. Total sugar content (mg/mg) = C × V/M, where C is the result calculated based on the standard curve (mg/mL); M is the actual weighed mass (mg); V is the total volume of the extract (mL).

#### 4.2.2. Chemical Composition and Molecular Weight Analysis

The total carbohydrate content was determined using the phenol sulfate method, with glucose as the standard. The UV–vis spectrum of the water solution of the sample (5 mg/mL) was measured on a multifunctional microplate reader (Beijing, China) in the wavelength range of 200–1000 nm. The homogeneity and molecular weights of the various fractions were measured using SEC-MALLS-RI spectroscopy.

The weight and number-average molecular weights (Mw and Mn) and polydispersity index (Mw/Mn) of various fractions in 0.1 M NaNO_3_ aqueous solution containing 0.02% NaN_3_ were measured on a DAWN HELEOS-II laser photometer equipped with two tandem columns (300 × 8 mm, Tokyo, Japan), which was held at 45 °C using a model column heater. The flow rate was 0.6 mL/min. A differential refractive index detector (Beijing, China) was simultaneously connected to determine the concentration of fractions and the dn/dc value. The dn/dc value of the fractions in 0.1 M NaNO_3_ aqueous solution containing 0.02% NaN_3_ was determined to be 0.141 mL/g. The data were acquired and processed using ASTRA6.1 (Beijing, China). The molecular weight is calculated as follows: *Mw* $=\frac{\sum wiMi}{\sum wi}$, $Mn=\frac{\sum niMi}{\sum ni}$, PDI $=\frac{Mw}{Mn}$, *Wi* is the mass fraction of the component, *ni* is the molar fraction of the component, and *Mi* is the molecular weight of the component.

#### 4.2.3. Monosaccharide Composition

A sample (5 mg) was hydrolyzed with trifluoroacetic acid (2 M) at 121 °C for 2 h in a sealed tube. The samples were dried using nitrogen. Methanol was added to wash, blow-dried, and methanol wash 2–3 times. The residue was re-dissolved in deionized water and filtered through a 0.22 μm microporous filter for measurement.

The sample extracts were analyzed by high-performance anion-exchange chromatography (HPAEC) on a CarboPac PA-20 anion-exchange column (3 × 150 mm; Dionex) using a pulsed amperometric detector (PAD; Dionex ICS 5000 +, Thermo, Newark, DE, USA). Flow rate, 0.5 mL/min; injection volume, 5 μL; solvent system A: (ddH_2_O), solvent system B: (0.1M NaOH), solvent system C: (0.1 M NaOH, 0.2 M NaAc); gradient program, volume ratio of solution A, B, C was 95:5:0 at 0 min, 85:5:10 at 26 min, 85:5:10 at 42 min, 60:0:40 at 42.1 min, 60:40:0 at 52 min, 95:5:0 at 52.1 min, 95:5:0 at 60 min. Data were acquired using an ICS5000+ (Beijing, China) and processed using Chromeleon 7.2 CDS (Beijing, China). The quantified data were output in Excel format.

#### 4.2.4. Fourier Transform Infrared (FT-IR) Analysis

Fourier-transform infrared (FT-IR) spectra of the polysaccharides were obtained using a spectrometer (Nicolet iZ-10, Beijing, China) at room temperature. The polysaccharide samples (1 mg) were mixed with KBr powder and then pressed into 1 mm pellets for FT-IR measurements in the range of 4000–400 cm^−1^.

#### 4.2.5. Methylation Analysis

The polysaccharide sample was dissolved in dimethyl sulfoxide (DMSO). The solution was methylated in DMSO/NaOH using CH_3_I. After complete methylation, the permethylated products were hydrolyzed with 2 mol/L TFA at 121 °C for 1.5 h, reduced with NaBD4, and acetylated with acetic anhydride for 2.5 h (100 °C). The acetates were dissolved in chloroform and analyzed using GC-MS on an Agilent 6890A-5975C instrument equipped with an Agilent BPX70 chromatographic column (30 m × 0.25 mm × 0.25 µm, SGE, Lingwood, Australia). High-purity helium (split ratio 10:1) was used as the carrier gas with an injection volume of 1 μL. Mass spectrometry analysis was performed at an initial temperature of 140 °C for 2.0 min, which was increased to 230 °C at a rate of 3 °C/min for 3 min. The scan mode was SCAN with a range of 50–350 *m*/*z*.

#### 4.2.6. Nuclear Magnetic Resonance (NMR) Analysis

The sample was dissolved in 0.5 mL D_2_O to obtain a final concentration of 40 mg/mL. 1D-NMR and 2D-NMR (1H-NMR, 13C-NMR, COSY, NOESY, HMBC, and HSQC) NMR spectra were recorded at 25 °C using a Bruker AVANCE NEO 500M spectrometer (Karlsruhe, Germany) operating at 500 MHz.

#### 4.2.7. Scanning Electronic Microscope (SEM) Analysis

The molecular morphologies of QSPS-1D were observed using a scanning electron microscope (SEM; Oberkochen, Germany). The samples were coated with a thin gold layer and placed on the substrate, and the images were then observed at a voltage of 1.0 kV with 500- and 10,000-fold magnification under high vacuum.

### 4.3. Screening of QSPS-1D In Vivo for Optimal Medicinal Concentrations

To determine the optimal drug concentration, LC50 tests were performed. In brief, 210 embryos of healthy wild-type (AB) zebrafish developing up to 6 hpf were selected and randomly assigned to six-well plates in the control (E3 culture) and QSPS-1D groups with different concentrations (25, 50, 100, 200, 400, and 800 μg/mL), with three parallels in each group (*n* = 30). Subsequently, they were transferred to the incubator for incubation, the culture medium was changed every day, and samples were taken until 96 hpf to determine the mortality rate, heart rate, malformation species, and malformation rate. The results showed an LC50 of 164.7 μg/mL for QSPS-1D on 96 hpf zebrafish larvae; therefore, subsequent tests were conducted using 20, 40, and 80 μg/mL (Figure S1).

### 4.4. Hepatoprotective Effect Evaluation

#### 4.4.1. Zebrafish Management and Model Building

Adult wild AB zebrafish were cultured in the laboratory of Guangxi Academy of Fisheries Science, with water temperature of 28–30 °C, pH 7.0–7.5, 14 h/10 h light–dark cycle, and fed three times a day (8:00, 12:00 and 18:00). To produce embryos, male and female zebrafish were paired 1:1 at night and isolated with a baffle; the baffle was removed the next day and spawned within 1 h of the light cycle. Later, healthy embryos were transferred to a solution containing methylene blue (0.3 ppm) 1xE3 and acclimatized in an artificial climate incubator (14 h/10 h light/dark) at 28.5 °C until 5 dpf before being used in experiments.

**Figure 9:**
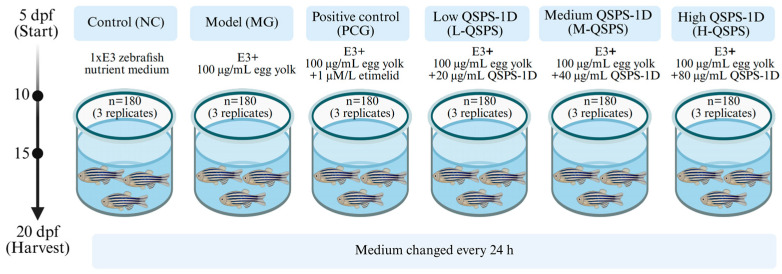
Zebrafish feeding schedule.

A total of 1080 healthy fish at 5 dpf were randomly divided into six groups: control (NC), model (MG), positive control (PCG), and high/medium/low QSPS-1D treatment groups (H-QSPS, M-QSPS, and L-QSPS), with three replicates per group (*n* = 180) (Figure 9). This study established a juvenile zebrafish NAFLD model by feeding high-fat egg yolk powder (containing 55.8% fat, 34.2% protein, and 17.2% saturated fatty acids) at 100 μg/mL for five consecutive days [51,52]. Zebrafish in the NC group were cultured in 1×E3 zebrafish nutrient medium. The MG, PCG, and polysaccharide groups were fed a nutrient solution supplemented with 100 μg/mL of egg yolk powder. The MG and polysaccharide groups received additional supplementation with etimelid (1 μM/L) and different concentrations of QSPS-1D (20, 40, and 80 μg/mL), respectively, during the high-fat diet. The culture medium was changed every 24 h. Fish were harvested 15 days post-hatching for the efficacy studies.

#### 4.4.2. Growth Performance and Biochemical Analysis

Thirty fish were randomly selected from each group for length and weight measurements. The activity levels of TG, TC, ALT, and AST in the liver were assayed according to the manufacturer’s instructions of commercially available kits.

#### 4.4.3. Quantitative Real-Time PCR Analysis

This study detected a total of 7 genes, including inflammatory cytokines (*il-1β*, *il-6*, and *tnf-α*), liver metabolism-related genes (*fasn*), and intestinal tight junction protein genes (*claudin-1*, *occludin*, and *zo-1*). The *actin* was used as an internal reference gene. Primer sequences were listed in Table 3. Total RNA was extracted using the TRIzol reagent. cDNA was synthesized using a reverse transcription kit B639252 (Sangon Biotech, Shanghai, China), followed by RT-qPCR analysis using an RT-PCR kit B630002 (Sangon Biotech). A 20 μL reaction system was employed, comprising 10 μL 2× Green-2-Go Mastermix, 0.4 μL forward/reverse primers (10 μM), 4 μL cDNA template, and 5.2 μL ddH_2_O. RT-qPCR analysis was performed using an ABI StepOnePlus instrument (ABI, California, USA) with the following program: 95 °C for 90 s; 40 cycles of 95 °C for 5 s, 60 °C for 15 s, and 72 °C for 20 s. The data were processed using the 2^−ΔΔCt^ method (*n* = 3).

#### 4.4.4. Histological and Immunohistological Analysis

Ten fish from each group were selected for morphological observation of their livers. The samples were fixed in 10% buffered formalin, embedded in paraffin, and sectioned into 5-μm slices using a cryostat. Liver tissue sections were stained with hematoxylin and eosin (H&E) and Oil Red O (ORO). Stained sections were examined using an optical microscope. Image software (java 8.0) was used to calculate the liver size and analyze the optical density (IOD) of lipid droplet deposition.

#### 4.4.5. Gut Microbiota Assessment

Genomic DNA was extracted from all samples using the CTAB method, and the purity and quality of the DNA were examined using agarose gel electrophoresis. Subsequently, the 16S rRNA gene in the V3-V4 region was amplified by PCR using universal primers, and the products were purified, quantified, and sequenced using the Illumina MiSeq platform. Data quality was assessed using FastQC (v0.11.9) and summarized using MultiQC software (v1.29). Initial QC was performed using Cutadapt to remove primers and discard all sequences that did not contain primer sequences. Further quality filtering and taxonomic identification were performed using DADA2. α- and β-diversity were analyzed using the QIME2 and R packages, respectively. Non-metric multidimensional scaling (NMDS) and principal coordinate analysis (PCoA) were performed on the Omicsmart bioinformatics platform: “http://www.omicshare.com/tools/” (accessed on 28 April 2025). Functional predictions were made using PICRUSt2 to predict the enrichment of ASVs in KEGG pathways.

### 4.5. Statistical Analysis

Data are presented as mean ± standard deviation (SD). Data analysis was performed using Prism 8.0. Differences between two or more groups were determined using one-way analysis of variance (ANOVA) and Tukey’s multiple comparison test. Statistical significance was set at *p* < 0.05.

## Figures and Tables

**Table 1:** Methylation analysis of QSPS-1D.

No.	Type of Linkage	PMAA	RT (min)	Mass Fragments (*m*/*z*)	Relative Molar Ratio (%)
1	t-Glc(p)	1,5-di-O-acetyl-2,3,4,6-tetra-O-methyl glucitol	13.935	71, 87, 102, 118, 129, 145, 161, 162, 205	12.32
2	4-Glc(p)	1,4,5-tri-O-acetyl-2,3,6-tri-O-methyl glucitol	17.336	87, 99, 102, 113, 118, 129, 131, 162, 173, 233	77.17
3	3,4-Glc(p)	1,3,4,5-tetra-O-acetyl-2,6-di-O-methyl glucitol	19.889	87, 88, 99, 113, 130, 173, 190, 233	1.08
4	4,6-Glc(p)	1,4,5,6-tetra-O-acetyl-2,3-di-O-methyl glucitol	21.134	85, 102, 118, 127, 159, 162, 201, 261	9.43

**Table 2:** 1H and 13C chemical shifts of QSPS-1D.

Code	Glycosyl Residues	Chemical Shifts (ppm)					
		H1/C1	H2/C2	H3/C3	H4/C4	H5/C5	H6/C6
A	→4)-α-D-Glcp-(1→	5.33	3.55	3.9	3.59	3.77	3.79,3.71
		99.8	71.54	73.34	76.89	71.25	60.55
B	α-D-Glcp-(1→	4.91	3.53	3.64	3.36	3.66	3.77
		98.26	71.19	72.9	69.33	72.71	59.38
C	→4,6)-α-D-Glcp-(1→	5.3	3.52	3.95	3.54	3.78	3.87
		100.1	71.73	73.28	77.01	70.91	69.33

**Table 3:** List of primers for RT-qPCR in zebrafish.

Genes	Primer	Sequence(5′-3′)
*fasn*	Forward	TTCTGTAACGTTGCCGGGAG
	Reverse	GGAAGGCCATACAGACCTGG
*il-1β*	Forward	CTGGTGTGTGACGACCTGCT
	Reverse	GCTGTGCTTCGTTGTCCTTG
*il-6*	Forward	CAGACCCAGACAGCCAACCT
	Reverse	GTCGCTCTGGTTCCTCATGT
*tnf-α*	Forward	GCTGACACCACGAGCATCCT
	Reverse	CAGGGTCATCGTTCGTTGGA
*claudin-1*	Forward	GAAGCGCCACAGGCTTTTTG
	Reverse	CCGAACTCATACCTCGCGTT
*occludin*	Forward	CTTGACACAGAGTATGAAACTGAAT
	Reverse	CTGTCGAGCTCTCGATCTGC
*zo-1*	Forward	GAGCAACGGCAGCCATAGAA
	Reverse	CTGCGGAGGATGCTGTCAT
*actin*	Forward	TGAGCAGGAGATGGGAACC
	Reverse	CAACGGAAACGCTCATTGC

## Data Availability

The data that support the findings of this study are available from the corresponding author, upon reasonable request.

## Supplementary Materials

The following supporting information can be downloaded at: https://www.mdpi.com/article/10.3390/md24050159/s1. Figure S1: Optimal dosage screening for QSPS-1D in vivo; Table S1: Molecular weight and distribution of neutral polysaccharides’ QSPS-1D.

## Abbreviations

The following abbreviations are used in this manuscript:

*il**-1β*	Interleukin-1β
*il-6*	Interleukin-6
*tnf-α*	Tumor Necrosis Factor-alpha
*fasn*	Fatty Acid Synthase
RNA	Ribonucleic acid
E3	Embryo medium (zebrafish culture medium)
dpf	days post-fertilization
hpf	hours post-fertilization
AST	Aspartate Aminotransferase
ALT	Alanine Aminotransferase
H&E	Hematoxylin and Eosin
LC50	Lethal Concentration 50%
Mw	Weight-average Molecular Weight
Mn	Number-average Molecular Weight
NAFLD	Nonalcoholic Fatty Liver Disease
NASH	Nonalcoholic Steatohepatitis
NMDS	Non-metric Multidimensional Scaling
NMR	Nuclear Magnetic Resonance
NOESY	Nuclear Overhauser Effect Spectroscopy
ORO	Oil Red O
PCoA	Principal Coordinate Analysis
PCR	Polymerase Chain Reaction
PICRUSt2	Phylogenetic Investigation of Communities by Reconstruction of Unobserved States 2
PMAA	Partially Methylated Alditol Acetate
RT-qPCR	Reverse Transcription Quantitative Real-Time PCR
HPLC	High-Performance Liquid Chromatography
HSQC	Heteronuclear Single Quantum Coherence (NMR)
IOD	Integrated Optical Density
HFD	High-Fat Diet
HMBC	Heteronuclear Multiple Bond Correlation (NMR)
HPAEC	High-Performance Anion-Exchange Chromatography
HPGFC	High-Performance Gel Filtration Chromatography
TC	Total Cholesterol
TG	Triglyceride
