# Supplementary material for: Structural Characterization of an α-D-glucan from Bellamya purificata and Its Protective Effects on Non-Alcoholic Fatty Liver Disease in Zebrafish
Source: Mar Drugs. 2026 Apr 30;24(5):159. doi: 10.3390/md24050159 (PMC13209108; doi:10.3390/md24050159)
Supplement: Supplementary file 1 [file marinedrugs-24-00159-s001.zip › Figure S1 Optimal dosage screening for QSPS-1D in vivo.pdf]

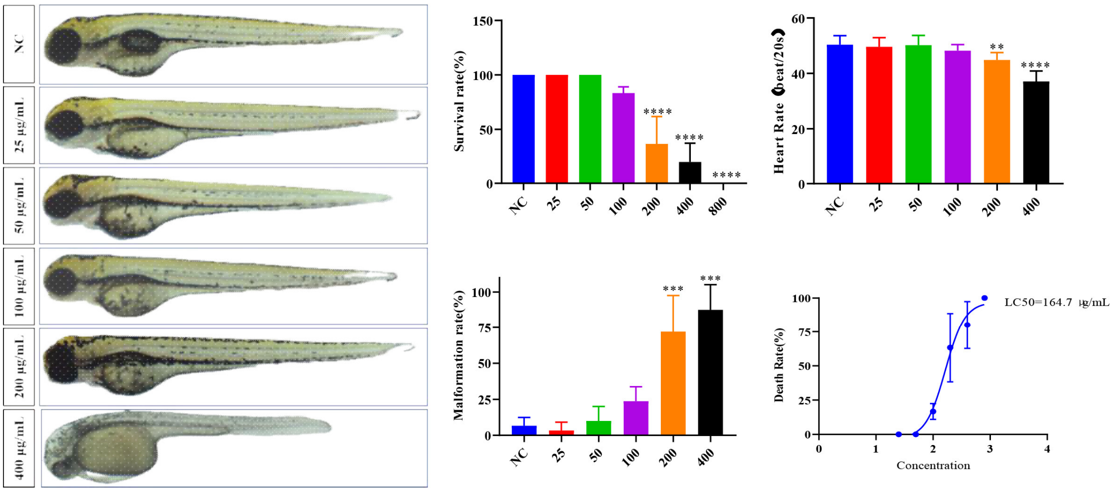

Figure S1. Optimal dosage screening for QSPS-1D in vivo

Note: * indicates $P < 0.05$; ** indicates $P < 0.01$; *** indicates $P < 0.001$; **** indicates $P < 0.0001$.
